# Resurgence of Respiratory Syncytial Virus Infection During COVID-19 Pandemic Among Children in Shanghai, China

**DOI:** 10.3389/fmicb.2022.938372

**Published:** 2022-07-01

**Authors:** Ran Jia, Lijuan Lu, Liyun Su, Ziyan Lin, Da Gao, Haiyan Lv, Menghua Xu, Pengcheng Liu, Lingfeng Cao, Jin Xu

**Affiliations:** ^1^Department of Clinical Laboratory, Children’s Hospital of Fudan University, Shanghai, China; ^2^School of Laboratory Medicine and Life Sciences, Wenzhou Medical University, Wenzhou, China; ^3^School of Laboratory Medicine, Bengbu Medical College, Bengbu, China; ^4^Shanghai Institute of Infectious Disease and Biosecurity, Fudan University, Shanghai, China

**Keywords:** respiratory syncytial virus—RSV, epidemiology, genotype, clinical features, COVID-19

## Abstract

Respiratory syncytial virus (RSV) is the most common pathogen causing acute lower respiratory tract infection (LRTI) in children. RSV usually peaks in winter and declines by early spring in China. The outbreak of coronavirus disease 2019 (COVID-19) was reported to bring changes to the transmission pattern of respiratory pathogens including RSV. Here in this paper, we analyzed RSV-positive nasopharyngeal aspirates from inpatients in the Children’s Hospital of Fudan University from October 2019 to October 2021 and compared the clinical features of the RSV-positive patients before and during COVID-19. We found an atypical upsurge of RSV infection in the late summer of 2021 after a major suppression in 2020. RSV B was the main subtype spreading among children throughout the study. Phylogenetic analysis revealed that all RSV A strains belonged to ON1 genotype and all RSV B strains were BA9 genotype. Deduced amino acid analysis displayed different substitutions in the RSV strains observed before and during COVID-19. Demographic analysis suggested that males and infants aged under 5 months were the main populations infected with RSV by gender and age, respectively. Less severe clinical outcomes were observed in patients during COVID-19 than before the pandemic, especially in RSV B-positive patients. Our findings described the epidemiological changes in RSV infection brought by COVID-19, which further underscored the importance of continuous surveillance of RSV in the shadow of COVID-19 at both local and global scales.

## Introduction

Respiratory syncytial virus (RSV) is the leading pathogen causing acute lower respiratory tract infection (LRTI) in the pediatric population, leading to almost 33.8 million episodes, 3.4 million hospitalizations, and 66,000–199,000 deaths among children under 5 years old (Di Mattia et al., 2021). RSV-induced LRTI in early childhood is a significant risk factor for the development of recurrent wheezing, asthma, and impaired lung function in later life (Young and Smitherman, 2021). Although some hopeful candidate vaccines and monoclonal antibodies are under development (PATH, 2021), there are yet no effective antiviral drugs or approved vaccines for RSV after 60 years of research (Jordan et al., 2021). A monoclonal antibody to the RSV F protein named palivizumab could reduce the rate and severity of RSV infections when administered preseasonally to children. But its use is limited to high-risk infants due to the high cost of medication (Boyoglu-Barnum et al., 2019). Therefore, the control of RSV transmission is still a tough problem globally.

RSV is an enveloped, single-stranded, negative-sense RNA virus. The RSV genome, which is around 15 kb, encodes at least 11 proteins, among which the attachment (G) protein and fusion (F) protein are key to mediating viral entry and inducing virus-neutralizing antibodies (Krishnamoorthy et al., 2012). The second hypervariable region (HVR2) of the G gene is highly genetically diverse and prone to be under selection pressure, which enabled it to be commonly used for the molecular classification of RSV (Lee et al., 2021). RSV has been classified as subgroups A and B based on genetic and immunological analyses, and further subclassified into at least 13 RSV A and 20 RSV B genotypes based on the sequences of the HVR2 of the G gene (Ma et al., 2021).

As a typical seasonal virus, RSV usually peaks in winter and declines by early spring in most temperate countries, with a median duration of 10–21 weeks (Staadegaard et al., 2021). However, the unexpected coronavirus disease 2019 (COVID-19) pandemic has exerted extraordinary changes to community behavior at a population level and remarkably restricted the prevalence of respiratory pathogens (Liu et al., 2021; Jia et al., 2022). However, along with the normalization of the COVID-19 epidemic control, an atypical reemergence of RSV was reported in various places (Agha and Avner, 2021; Foley et al., 2021; Government of Western Australia, 2021; Ujiie et al., 2021; Camporesi et al., 2022; Eden et al., 2022; Saravanos et al., 2022). Hence, we conducted this research to determine if RSV also resurged in Shanghai in the setting of COVID-19 pandemic and to demonstrate the differences between the resurgence with the previous RSV season.

## Materials and Methods

### Patients and Sample Collection

A total of 1,526 out of 18,371 inpatients in the Children’s Hospital of Fudan University were defined as RSV-positive after routine screening for respiratory viruses in the clinical laboratory from December 2018 to February 2022 based on the medical records in the hospital. The routine respiratory virus screening (including RSV, adenovirus, influenza A and B viruses, parainfluenza virus, human rhinoviruses, and human metapneumovirus) was performed using a 24-capillary Applied Biosystems^™^ 3500×L Dx Genetic Analyzer (Thermo Fisher, United States), which is an automatic clinical detection system based on PCR and capillary electrophoresis.

For further genotyping, 403 nasopharyngeal aspirates were collected randomly from the RSV-positive inpatients from October 2019 to October 2021. Severe cases were defined by experienced clinicians according to the World Health Organization (WHO)‘s latest definitions of severe LRTIs (Vanker et al., 2017; Jia et al., 2022). Demographic information and medical data for each patient were collected from electronic records in the hospital.

All experiments in the study were carried out following relevant guidelines and regulations. We obtained informed consent from each child’s parents or guardians before enrollment. This study was approved by the Ethics Committee of the Children’s Hospital of Fudan University in February 2020 (Approval Number: 202027).

### RSV Genotyping

The HVR2 of the G gene of the 403 RSV samples was then amplified and sequenced for subtyping. The reverse transcription was performed using the PrimeScript RT-PCR kit (Takara, Japan). The amplification step was performed using the Premix Taq kit (Takara, Japan) with RSV A primers (RSVA-GF 5′-TATGCAGCAACAATCCAACC-3′ & RSVA-F1-R 5′-CAACTCCATTGTTATTTGCC-3′) and RSV B primers (RSVB-GF 5′-GCAGCCATAATATTCATCATCTCT-3′ and RSVB-GR 5′-TGCCCCAGRTTTAATTTCGTTC-3′). The amplification products were 531 and 800 bp for RSV A and RSV B, respectively. After the amplification, the products were subjected to Sanger sequencing by Sangon Biotech Co., Ltd., China. The sequence data were then compared to the reference sequences of RSV strains on GenBank for genotyping.

### Phylogenetic and Amino Acid Substitution Analysis

The sequences of the HVR2 of the G gene derived in our study were aligned with the reference sequences obtained from GenBank using the MUSCLE program. The phylogenetic trees were constructed using the neighbor-joining method and branch supported with 1,000 bootstrap iterations. The deduced amino acid sequences were translated with standard genetic code and mutations were determined through comparisons of the strains with the corresponding prototype strains. The phylogenetic and amino acid substitution analyses were all implemented using MEGA software (version 11).

### Statistical Analysis

A chi-square test or Fisher’s exact test was used for the comparison of proportions for categorical variables. An unpaired Student’s t test was used to test the differences in quantitative variables with a normal distribution. Otherwise, a Mann–Whitney *U* test was used. Univariate Poisson regression analysis was initially used to investigate the associations of RSV-positive severe LRTI cases with season at onset, RSV subtype, gender, age, gestational age at birth, co-infections with other respiratory viruses, underlying diseases, and laboratory findings. Then multivariate Poisson regression analysis was performed, incorporating all factors with *p* < 0.1 in the univariate analysis. Relative risk (RR) and 95% confidence interval (CI) were reported. Poisson regression analyses were performed using IBM SPSS Statistics software (version 23), while the other analyses were performed using GraphPad Prism software (version 6). Two-sided *p*-values of <0.05 were considered significant.

## Results

### Epidemiological Surveillance of RSV

**Figure 1:**
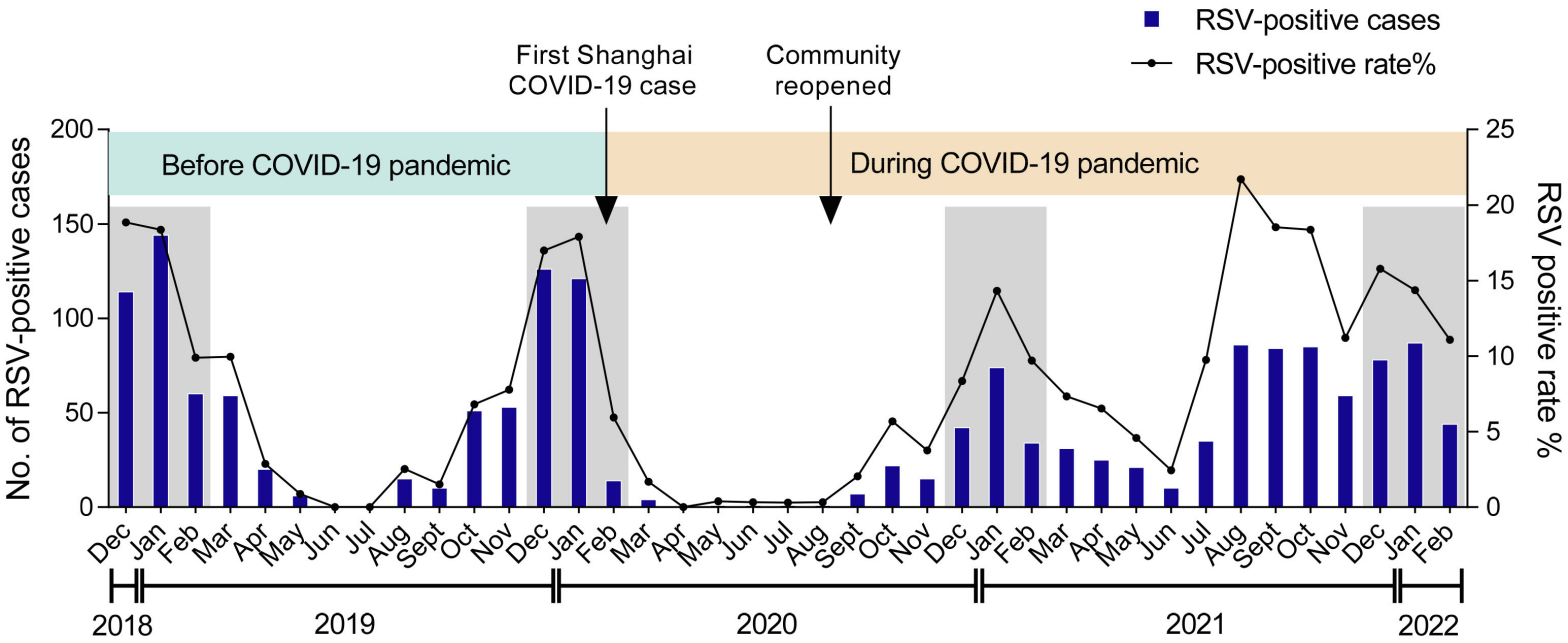
Monthly distribution of RSV among children. The numbers and positive rate of RSV-positive inpatients admitted to the Children’s Hospital of Fudan University from December 2018 to February 2022 were displayed. Arrows labeled the time points when the first COVID-19 case was reported in Shanghai and when the community was reopened. The winter seasons (December–February) are framed by gray rectangles.

A total of 1,526 out of 18,371 inpatients were tested positive for RSV in the Children’s Hospital of Fudan University from December 2018 to February 2022. Given that the first COVID-19 case in Shanghai was reported on Jan 20, 2020, followed by a national COVID-19 pandemic (Zhang et al., 2020), we divided the RSV seasons into two phases—before COVID-19 pandemic and during COVID-19 pandemic (Figure 1). Both RSV-positive rate and case numbers typically peaked in the winter months (December to February) before COVID-19, but sharply declined because of the restriction policies against COVID-19 and kept at a low level until the autumn months (September to November) of 2020, when the community reopened. After that, RSV infections displayed a gradual increment, peaking in the middle winter (January) of 2020/2021 (14.3%). Afterward, RSV-positive rate reached its highest percentage positive rate (21.7%) throughout the study in the late summer (August) of 2021, followed by an unusual decrease in the autumn. These data suggested that RSV showed an abnormal transmission pattern during COVID-19 pandemic.

### Genotyping of RSV

To figure out the prevailing genotypes of RSV, we collected and genotyped a total of 403 RSV-positive samples from October 2019 to October 2021 in the Children’s Hospital of Fudan University, among which 170 samples were collected before COVID-19 pandemic (October 2019 ~ January 2019) and the rest 233 samples were collected during the pandemic (November 2020 ~ October 2021).

**Figure 2:**
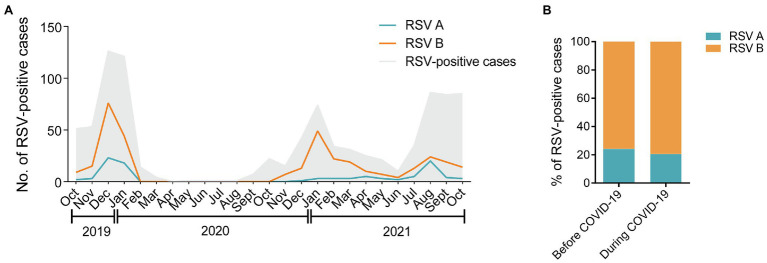
Distribution of RSV A and B subgroups among children before and during COVID-19. **(A)** The monthly distribution of RSV A and B from October 2019 to October 2021. The gray shadow represents the total number of all the RSV-positive inpatients admitted to the hospital. The blue and orange lines represent the numbers of RSV A- or B-positive inpatients enrolled in the study, respectively. **(B)** The proportions of RSV A- or B-positive inpatients enrolled in the study before and during COVID-19.

As shown in Figure 2A, RSV B was the major subtype transmitting among children in the three consecutive RSV seasons throughout the study. During the COVID-19 pandemic, the number of RSV A-positive cases showed an increase in August 2021 after a long-time suppression, followed by a rapid reduction in September 2021. The percentages of RSV A and B were comparable before and during COVID-19 (24.2% vs. 20.6% for RSV A; 75.8% vs. 79.4% for RSV B, *p =* 0.3587; Figure 2B). No dual infections of RSV A and B were detected in the 403 samples.

**Figure 3:**
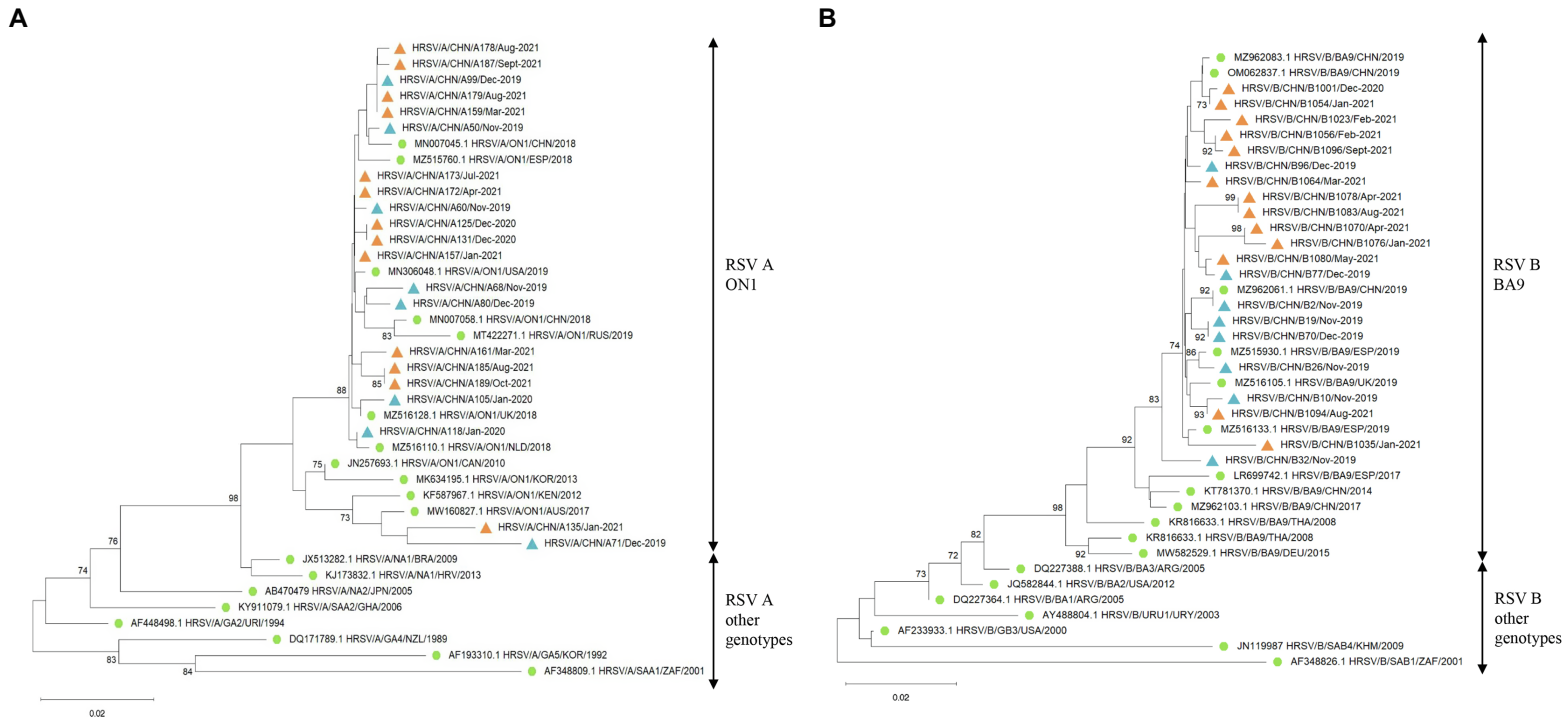
The G gene phylogenetic trees of RSV A and B strains. The phylogenetic trees of RSV A strains **(A)** and RSV B strains **(B)** derived in the study during 2019–2021 based on the HVR2 of the G gene. The phylogenetic trees were constructed using neighbor-joining method with 1,000 bootstrap replicates. Bootstrap values > 70% were shown. Reference genotypes downloaded from the GenBank were labeled with green dots. RSV strains derived in the study were labeled with blue triangles before COVID-19 and orange triangles during the pandemic.

Phylogenetic analysis of the HVR2 of the G gene indicated that all RSV A strains belonged to the ON1 genotype (Figure 3A) and all RSV B strains were BA9 genotype (Figure 3B). The GenBank accession numbers of the sequences derived in our study are listed in Supplementary Table 1. The G gene phylogeny did not reveal distinct lineages of RSV strains during COVID-19. The RSV strains before and during COVID-19 derived in our study were genetically close to the reference strains observed during 2018 to 2019. In addition, RSV B strains before COVID-19 were more genetically close to the strains observed in Spain, the United Kingdom, and China in 2019, while RSV B strains during the pandemic were mainly close to the Chinese strains in 2019.

### Deduced Amino Acid Analysis of RSV

The deduced amino acid sequences (according to the HVR2 of the G protein) of RSV A and RSV B strains were aligned and compared with the RSV A ON1 prototype (GenBank accession no. JN257693) and RSV B BA9 prototype (GenBank accession no. DQ227395), respectively (Supplementary Figure 1A). Compared with the ON1 prototype, most of the ON1 strains in our study shared H258Q and H266L substitutions. Fixed sets of genetic alterations of V225A/I243T and E286K/P300Q were more commonly observed in the strains collected before COVID-19 pandemic. Other substitutions like E257K, L274P, L298P, and Y304H were distributed in strains collected both before and during COVID-19.

All BA9 strains derived in our study harbored I279T, T288I, and F295S substitutions, while T252I, P262T, L265S, and T310I existed in most of the strains (Supplementary Figure 1B). A fixed combination of P229L/S255L/E290K was more commonly observed in strains collected before COVID-19, while A269V, T274A, and T300I were more frequently seen in strains discovered during COVID-19.

### Demographic and Clinical Features of RSV-Positive Cases

Male patients were the main group infected with RSV throughout the study, accounting for 61.8% before COVID-19 and 59.7% during COVID-19 (Table 1). The median age of the patients increased from 2 m before COVID-19 to 4 m during COVID-19 (*p =* 0.0329). Patients aged 0 ~ 5 m were the main group infected with RSV (65.3% before COVID-19 vs. 58.8% during COVID-19). Further classification revealed that there were fewer patients aged < 1 m during COVID-19 (23.2%) compared with that before the pandemic (33.5%; *p* = 0.0216). RSV-positive children during COVID-19 showed a higher rate of jaundice, but lower rates of rhinorrhea, cough, tachypnea, and respiratory failure than in the previous RSV season. Notably, the percentage of severe LRTI cases was 10.6% in patients before COVID-19 and 2.1% during COVID-19 (*p =* 0.0003). The laboratory findings showed that RSV-positive children during COVID-19 showed slightly lower neutrophil numbers and serum creatinine (Cr) levels, but higher procalcitonin (PCT) values.

**Table 1:** Demographic and clinical features of RSV-positive patients before and during COVID-19.

	Before COVID-19	During COVID-19	*p* Value
Total case number	170	233	
**Gender**			
Male	105(61.8%)	139(59.7%)	0.6689
Female	65(38.2%)	94(40.3%)	
**Age**			
<1 m	57(33.5%)	54(23.2%)	0.0216*
1–2 m	38(22.4%)	58(24.9%)	0.5545
3–5 m	16(9.4%)	25(10.7%)	0.6656
6–11 m	17(10%)	24(10.3%)	0.9215
12–23 m	17(10%)	28(12%)	0.5255
2–4y	23(13.5%)	35(15%)	0.6734
5–18y	2(1.2%)	9(3.9%)	0.1852
**Gestational age at birth, weeks**			
<34	15(8.8%)	13(5.6%)	0.2059
34–36	12(7.1%)	10(4.3%)	0.2272
≥37	143(84.1%)	210(90.1%)	0.0706
**Clinical manifestations**			
Fever	38(22.4%)	59(25.3%)	0.4911
Rhinorrhea	30(17.6%)	25(10.7%)	0.0457*
Cough	133(78.2%)	143(61.4%)	0.0003*
Wheeze	26(15.3%)	29(12.4%)	0.4108
Jaundice	4(2.4%)	17(7.3%)	0.0479*
Tachypnea	15(8.8%)	7(3%)	0.0111*
Respiratory failure	12(7.1%)	3(1.3%)	0.0059*
Severe LRTI	18(10.6%)	5(2.1%)	0.0003*
Hospitalization duration, days, median (IQR)	10(7–15)	10(7–16)	0.0924
**Laboratory findings, median (IQR)**			
WBC, ×10^9/L	8.4(6.48 ~ 11.15)	8.01(5.92 ~ 10.57)	0.1419
Lymphocytes, ×10^9/L	4.05(2.9 ~ 5.58)	4.23(2.83 ~ 5.67)	0.6566
Neutrophils, ×10^9/L	2.61(1.68 ~ 4.16)	2.205(1.38 ~ 3.23)	0.0104*
Eosinophils, ×10^9/L	0.1(0.01 ~ 0.24)	0.14(0.02 ~ 0.3)	0.0705
CRP, mg/L	1.13(0.25 ~ 4.7)	1.55(0.25 ~ 4)	0.7996
PCT, ng/dl	0.09(0.06 ~ 0.18)	0.36(0.2 ~ 0.5)	< 0.0001*
IL-6, pg/ml	16.95(5.16 ~ 62.9)	12.265(4.42 ~ 31.29)	0.2194
ALT, U/L	21.4(14.75 ~ 32.18)	21.7(15.17 ~ 37.08)	0.3364
AST, U/L	39.2(31.1 ~ 54.18)	43.1(33.3 ~ 63.48)	0.0580
Urea, mmol/L	2.7(2 ~ 3.6)	2.81(2.16 ~ 3.6)	0.3445
Cr, μmol/l	24(20.75 ~ 30)	22.45(19.15 ~ 26.45)	0.0140*
**Underlying diseases**	85(50.0%)	105(45.1%)	0.3270
Cardiovascular diseases	21(12.4%)	31(13.3%)	–
Hepatobiliary diseases	12(7.1%)	24(10.3%)	–
Metabolic diseases	4(2.4%)	2(0.9%)	–
Malignant tumor	2(1.2%)	2(0.9%)	–
Immunodeficiencies	–	2(0.9%)	–
Bronchopulmonary dysplasia	2(1.2%)	2(0.9%)	–
**Co-infections**			
Rhinovirus	4(2.4%)	6(2.6%)	–
Parainfluenza virus	2(1.2%)	3(1.3%)	–
Bocavirus	–	2(0.9%)	–
Metapneumovirus	8(4.7%)	–	–

LRTI, lower respiratory tract infection; IQR, interquartile range; CRP, C-reactive protein; PCT, procalcitonin; ALT, alanine transaminase; AST, aspartate aminotransferase; Cr, creatinine.

* *p* < 0.05.

After involvement of RSV subtypes, we found that the percentages of male patients between RSV A and B were similar before COVID-19 (61.5% for RSV A vs. 61.8% for RSV B, *p* = 0.9736), but different during COVID-19 (47.1% for RSV A vs. 63.2% for RSV B, *p =* 0.0380; Table 2). We did not find meaningful differences in the age groups either among RSV subtypes or in different phases, except for the 12–23 m group, where the percentage of RSV A-positive patients before COVID-19 was smaller than that of the patients during COVID-19 (0 vs. 11.8%, *p =* 0.0342). There were more full-term birth children during COVID-19 (91.8%) compared with that before the pandemic (83.2%) among RSV B-positive children (*p* = 0.0208). As for clinical features, RSV A-positive patients during COVID-19 were mainly comparable with those before the pandemic, except for a longer hospitalization duration and higher PCT values. The clinical characteristics of the patients infected with RSV B during COVID-19 resembled those of the total patients during COVID-19 as we observed in Table 1. We observed no significant difference in the clinical features between RSV A- and RSV B-positive patients either before or during COVID-19. Overall, these data suggested a decrease in the disease severity among RSV-positive patients during COVID-19, especially for patients infected with RSV B.

**Table 2:** Demographic and clinical features of RSV A- or RSV B-positive patients before and during COVID-19.

	RSV A			RSV B		
	Before COVID-19	During COVID-19	*p* Value	Before COVID-19	During COVID-19	*p* Value
Total case number	39	51		131	182	
**Gender**						
Male	24(61.5%)	24(47.1%)	0.1724	81(61.8%)	115(63.2%)	0.8069
Female	15(38.5%)	27(52.9%)		50(38.2%)	67(36.8%)	
**Age**						
<1 m	13(33.3%)	11(21.6%)	0.2111	44(33.6%)	43(23.6%)	0.0523
1–2 m	9(23.1%)	14(27.5%)	0.6373	29(22.1%)	51(28.0%)	0.2390
3–5 m	5(12.8%)	9(17.6%)	0.5313	11(8.4%)	9(4.9%)	0.2180
6–11 m	3(7.7%)	2(3.9%)	0.7569	14(10.7%)	22(12.1%)	0.7016
12–23 m	0(0%)	6(11.8%)	0.0342*	17(13.0%)	22(12.1%)	0.8142
2–4y	8(20.5%)	7(13.7%)	0.3919	15(11.5%)	28(15.4%)	0.3186
5–18y	1(2.6%)	2(3.9%)	0.7222	1(0.8%)	7(3.8%)	0.1796
**Gestational age at birth, weeks**						
<34	4(10.3%)	6(11.8%)	0.9102	11(8.4%)	7(3.8%)	0.0880
34–36	1(2.6%)	2(3.9%)	0.8127	11(8.4%)	8(4.4%)	0.1436
≥37	34(87.2%)	43(84.3%)	0.7015	109(83.2%)	167(91.8%)	0.0208*
**Clinical manifestations**						
Fever	10(25.6%)	15(29.4%)	0.6923	28(21.4%)	44(24.2%)	0.5612
Rhinorrhea	3(7.7%)	4(7.8%)	0.7109	27(20.6%)	22(12.1%)	0.0407*
Cough	27(69.2%)	30(58.8%)	0.3100	106(80.9%)	113(62.1%)	0.0003*
Wheeze	4(10.3%)	5(9.8%)	0.7767	22(16.8%)	24(13.2%)	0.3739
Jaundice	1(2.6%)	4(7.8%)	0.5359	3(2.3%)	13(7.1%)	0.0963
Tachypnea	5(12.8%)	2(3.9%)	0.2441	10(7.6%)	5(2.7%)	0.0459*
Respiratory failure	2(5.1%)	1(2%)	0.8127	10(7.6%)	2(1.1%)	0.0075*
Severe LRTI	4(10.3%)	2(3.9%)	0.4428	14(10.7%)	3(1.6%)	0.0012*
Hospitalization duration, days, median (IQR)	10(7 ~ 16)	13(10 ~ 19)	0.0211*	10(7 ~ 15)	8(6 ~ 14)	0.8430
**Laboratory findings, median (IQR)**						
WBC, ×10^9/L	8.7(7.4 ~ 10.5)	7.62(5.7 ~ 9.8)	0.1394	8.4(6.3 ~ 11.3)	8.17(5.9 ~ 10.3)	0.3454
Lymphocytes, ×10^9/L	4.1(2.3 ~ 5.1)	3.56(2.3 ~ 4.7)	0.6742	4(3.1 ~ 5.8)	4.305(3.1 ~ 5.8)	0.4138
Neutrophils, ×10^9/L	3.66(1.8 ~ 5.6)	2.235(1.7 ~ 4.3)	0.2002	2.335(1.6 ~ 3.8)	2.125(1.4 ~ 2.9)	0.0246*
Eosinophils, ×10^9/L	0.02(0 ~ 0.2)	0.05(0 ~ 0.2)	0.3455	0.11(0 ~ 0.2)	0.15(0 ~ 0.3)	0.0630
CRP, mg/L	4(0.3 ~ 14)	1.02(0.3 ~ 4)	0.1637	0.695(0.3 ~ 4)	1.92(0.9 ~ 7.9)	0.2449
PCT, ng/dl	0.1(0.1 ~ 0.2)	0.17(0.1 ~ 0.5)	0.0021*	0.09(0.1 ~ 0.2)	0.37(0.2 ~ 0.5)	< 0.0001*
IL-6, pg/ml	11.63(3.7 ~ 41.4)	14.27(6.9 ~ 39.7)	0.6044	17.82(6.8 ~ 66.3)	11.87(3.8 ~ 29)	0.0809
ALT, U/L	21.1(13.5 ~ 37.2)	20.42(14.6 ~ 34.1)	0.9494	21.7(14.8 ~ 31.4)	22.7(15.3 ~ 37.1)	0.2333
AST, U/L	38.4(29.6 ~ 70.4)	47.6(32.7 ~ 67.1)	0.2960	40.4(32 ~ 51.1)	42.41(33.3 ~ 63.1)	0.1266
Urea, mmol/L	3.2(2.4 ~ 4.2)	2.82(2.3 ~ 3.8)	0.5437	2.6(1.8 ~ 3.4)	2.705(2.1 ~ 3.6)	0.1665
Cr, μmol/l	25(21 ~ 31)	24.4(20.6 ~ 27.8)	0.2826	24(20 ~ 29)	22(19 ~ 26)	0.0306*
**Underlying diseases**	21(53.8%)	29(56.9%)	0.7753	64(48.9%)	76(41.8%)	0.2129
Cardiovascular diseases	7(17.9%)	10(19.6%)	–	14(10.7%)	21(11.5%)	–
Hepatobiliary diseases	1(2.6%)	7(13.7%)	–	11(8.4%)	17(9.3%)	–
Metabolic diseases	2(5.1%)	–	–	2(1.5%)	2(1.1%)	–
Malignant tumor	1(2.6%)	–	–	1(0.8%)	2(1.1%)	–
Immunodeficiencies	–	1(2%)	–	–	1(0.5%)	–
Bronchopulmonary dysplasia	–	–		2(1.5%)	2(1.1%)	–
**Co-infections**						
Rhinovirus	1(2.6%)	1(2%)	–	3(2.3%)	5(2.7%)	–
Parainfluenza virus	–	–	–	2(1.5%)	3(1.6%)	–
Bocavirus	–	–	–	–	2(1.1%)	–
Metapneumovirus	3(7.7%)	–	–	5(3.8%)	–	–

* *p* < 0.05.

### Multivariate Analysis on Disease Severity

To further look for the causes of the decrease in disease severity of RSV infection during COVID-19, we performed Poisson regression analysis on the severe LRTI cases collected in our study (Supplementary Table 2). All factors showing *p* < 0.1 in the univariate analysis were considered for inclusion in the multivariate analysis. Three factors, including gestational age at birth, underlying diseases, and neutrophil counts were included in the multivariate analysis adjusted by before/during COVID-19. However, none of these factors showed significant associations with severe LRTI cases.

## Discussion

In this study, we observed an unusual increase of the RSV detection rate in the summer of 2021 in Shanghai. Interestingly, the abnormal reemergence of RSV during COVID-19 was also observed during the summer months in both northern hemisphere countries including Japan, America, Canada, Israel, Italy (Public Health Agency of Canada, 2021; Ujiie et al., 2021; Weinberger Opek et al., 2021; Camporesi et al., 2022; The US Centers for Disease Control and Prevention, 2022), and southern hemisphere countries including Australia and New Zealand (Agha and Avner, 2021; Anglemyer et al., 2021; Foley et al., 2021, 2022; Dolores et al., 2022; Saravanos et al., 2022). Hence, our findings reflected a global phenomenon. The factors contributing to the “summer peak” of RSV in the post-COVID-19 phase were complicated, possibly including relaxation of restriction measures, increased social activities of children, extensive respiratory virus testing to rule out COVID-19, and possible changes in the environmental resistance of RSV strains. Continuous surveillance of RSV infection at both local and global scales is needed to provide more clues to explain the phenomenon.

RSV-A ON1 genotype and RSV B BA genotype have become the two predominant strains globally since their emergence in 2010 and 1999, respectively (Trento et al., 2003; Eshaghi et al., 2012). In China, ON1 became the major RSV A genotype during 2013–2015 (Song et al., 2017), while BA9 took the dominance during 2008–2014 (Chen et al., 2021). In our study, all RSV A strains were ON1 and all RSV B strains were BA9 either before or during COVID-19, which was consistent with the reports from other places of China and other countries in recent years (Chen et al., 2021; Lee et al., 2022; Sun et al., 2022). RSV A and B were reported to take dominance alternately, with a major prevalent pattern of “BBAA” most of the time (Luo et al., 2020; Sun et al., 2022). But RSV B is the main prevailing subtype in our study, although there was a co-circulation of both RSV A and B. Differently, a study in Australia showed that there was an overwhelming predominance of RSV A (>95% cases) from late 2020 to early 2021 and all of the RSV A viruses belonged to ON1-like genotype (Eden et al., 2022). Moreover, they found two genetically distinct RSV A clades circulating cryptically, which did not cluster with any other RSV A viruses sampled nationally or internationally to date for unknown reason (Eden et al., 2022). We did not observe distinct RSV A linages during COVID-19 in our data, possibly due to the small number of RSV A-positive cases. But it is interesting that although their prevailing subtype was different from ours, we both observed a similar summer upsurge of RSV, suggesting that people’s behavioral changes brought by COVID-19-associated nonpharmaceutical interventions (NPIs), e.g., strict quarantine policies, limited social activities, school closures and reopenings seemed to exert more effect on RSV transmission pattern than the virus properties.

Our study indicated that the severity of the delayed RSV seasons during COVID-19 in Shanghai was decreased, as evidenced by less frequent respiratory failure and severe LRTI cases than before, which was similar with the studies conducted in Australia and France, characterized by fewer hospitalizations and ICU admissions (Fourgeaud et al., 2021; Foley et al., 2022; Saravanos et al., 2022). However, the impacts of the delayed RSV season on patients might vary in different settings. For instance, studies in Italy indicated that the severity of RSV-associated disease was comparable with the previous season according to the respiratory support and PICU admissions (Stera et al., 2021; Camporesi et al., 2022). Also, a study in the United States of America (USA) indicated more severe RSV-related diseases in infants during COVID-19 pandemic (Agha and Avner, 2021). As for explanations, the studies from Australia tended to attribute milder clinical presentations during COVID-19 to the lowered threshold for respiratory virus testing (Sullivan et al., 2020; Foley et al., 2022; Saravanos et al., 2022), while the study from the USA proposed that the increased severity might be associated with diminished immunity from a lack of exposure to RSV in the previous season (Agha and Avner, 2021). According to the multivariate analysis in our study, we did not find risk factors for severe LRTI cases among the widely accepted predictors for RSV-induced severe cases including age, gestational age at birth, underlying diseases and co-infections (Malosh et al., 2017; Ghazaly and Nadel, 2018; Lee and Goh, 2021), which prompted us to look for clues in people’s behavioral changes during COVID-19. In the setting of COVID-19, parents in China tended to take their children, particularly infants, into hospitals immediately once they showed signs of respiratory infection to rule out COVID-19, rather than waiting for a self-healing as most parents did before the pandemic. Moreover, the government has deployed more personnel and resources to facilitate the diagnosis and treatment of respiratory diseases, leading to better care of the patients. Hence, we assume that the timely diagnosis and sufficient medical resources might contribute to the decreased RSV-associated disease severity during COVID-19, which deserves further assessment and exploration.

Apart from the external factors, the genetic changes of RSV themselves might also play a role in the clinical outcomes of RSV-infected patients during COVID-19. In our study, we found some amino acid substitutions which were more frequently seen in RSV B BA9 strains during COVID-19 than before the pandemic, including A269V, T274A, and T300I. As reported, certain alterations could change the virulence of RSV. For instance, a study from China reported that the severity score was lower in RSV A-positive cases with a set of 5 substitutions (T113I, V131D, N178 G, H258Q, and H266 L) of G gene compared with other RSV A-positive cases, suggesting a possible causative relationship (Li et al., 2020). Also, a study from the Netherlands found that 8 substituted amino acids (S102F, K216N, P256S, H258Y, S270Y, E271K, P300S, and T306I) of G gene were associated with higher disease severity with a more frequent need for extra oxygen (Vos et al., 2019). The mechanism of how these amino acid alterations affect the outcomes of the disease is still unclear. We did not find the above-mentioned substitutions in our results, but it deserves further assessment about the role of the genetic changes of RSV strains in our study or other unknown changes in RSV genome in the changed clinical features during the pandemic.

There are some limitations of our study. First, the period and number of the samples collected were not enough to show more comprehensive and precise epidemiological features of RSV before and during COVID-19. Moreover, no dual infections of RSV A and B were detected in the 403 samples, possibly due to the limited sample number and the unusually low level of RSV A-positive cases from February 2020 to July 2021. Second, as with other similar studies, the medical records we acquired usually could not fully reflect the overall conditions of the patients, leading to possible deviations in our analyses. Last but not least, since we only focused on RSV in this study, we were unable to comprehensively assess the epidemiological features of other common respiratory pathogens including human rhinoviruses, influenza A and B viruses, and parainfluenza virus before and during COVID-19, which will be a promising topic in our future study.

Conclusively, our study displayed epidemiological features and phylogenetic information of RSV before and during COVID-19 in Shanghai. It is clear that COVID-19 affected the transmission pattern of RSV since 2020 and the patients in our study showed certain demographic and clinical changes in the context of COVID-19. These findings provides a timely warning to the countries suffering from COVID-19 that other respiratory viruses, such as RSV, still deserve full attention to avoid unusual rebound and unexpected impacts, and further necessitates the continuous surveillance of the epidemiological and evolutionary dynamics of RSV both spatially and temporally.

## Data Availability Statement

The original contributions presented in the study are included in the article/Supplementary Material, further inquiries can be directed to the corresponding author.

## Ethics Statement

This study was approved by the Ethics Committee of the Children’s Hospital of Fudan University in February 2020 (Approval Number: 202027). Written informed consent to participate in this study was provided by the participants’ legal guardian/next of kin.

## Author Contributions

RJ and JX conceived and designed the experiments, analyzed the data, and wrote the paper. RJ, LL, LS, MX, PL, and LC collected the nasopharyngeal aspirates from patients. RJ, ZL, DG, and HL collected the clinical data of patients. RJ, LL, ZL, DG, and HL performed the experiments. All authors contributed to the article and approved the submitted version.

## Funding

This work was supported by grants from the Key Development Program of Children’s Hospital of Fudan University (grant no. EK2022ZX05).

## Conflict of Interest

The authors declare that the research was conducted in the absence of any commercial or financial relationships that could be construed as a potential conflict of interest.

## Publisher’s Note

All claims expressed in this article are solely those of the authors and do not necessarily represent those of their affiliated organizations, or those of the publisher, the editors and the reviewers. Any product that may be evaluated in this article, or claim that may be made by its manufacturer, is not guaranteed or endorsed by the publisher.
