# Supplementary material for: Resurgence of Respiratory Syncytial Virus Infection During COVID-19 Pandemic Among Children in Shanghai, China
Source: Front Microbiol. 2022 Jul 1;13:938372. doi: 10.3389/fmicb.2022.938372 (PMC9298468; doi:10.3389/fmicb.2022.938372)
Supplement: Supplementary file 1 [file Data_Sheet_1.docx]

***Supplementary Material***

**
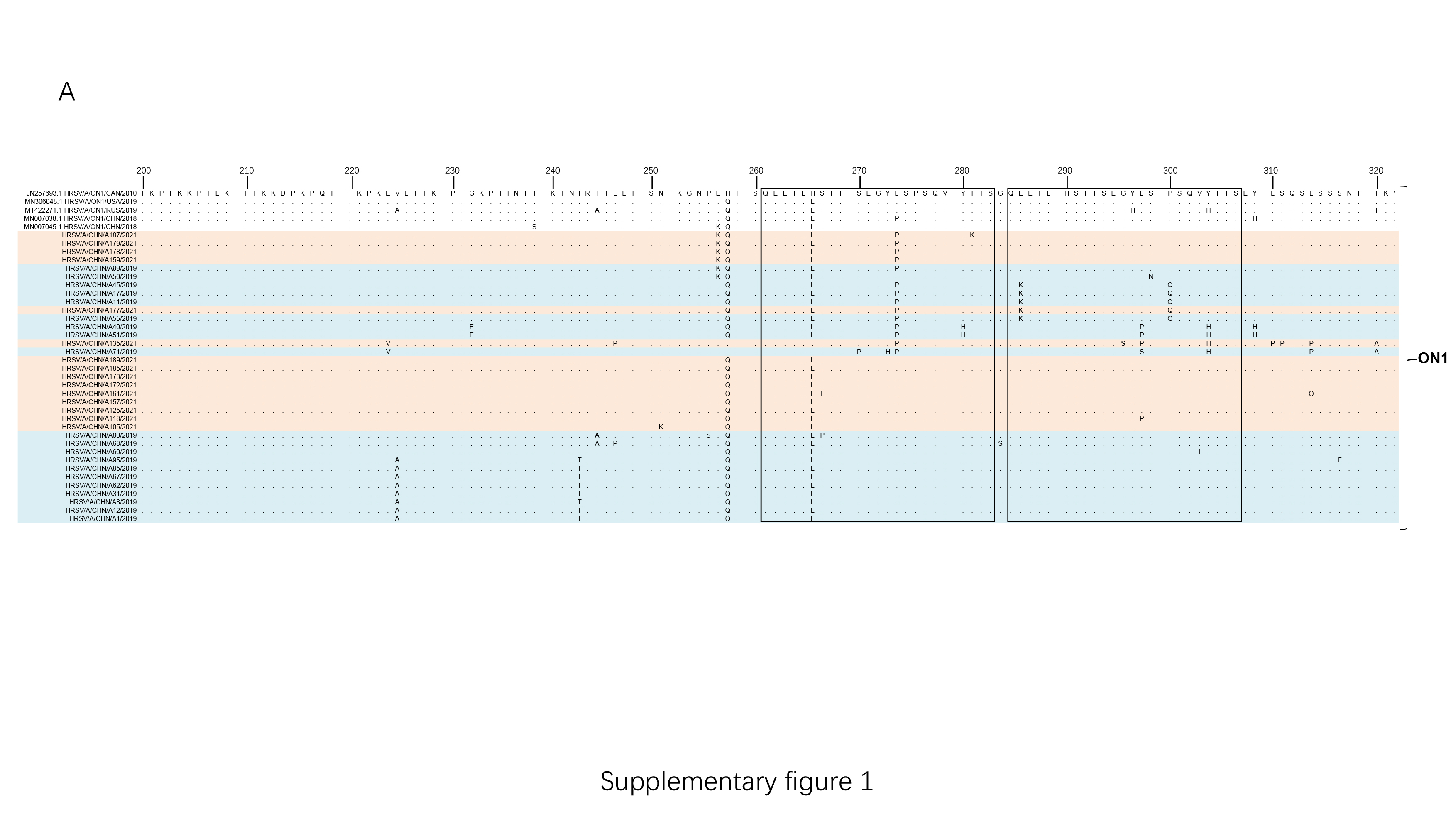
Supplementary Figures and Tables**


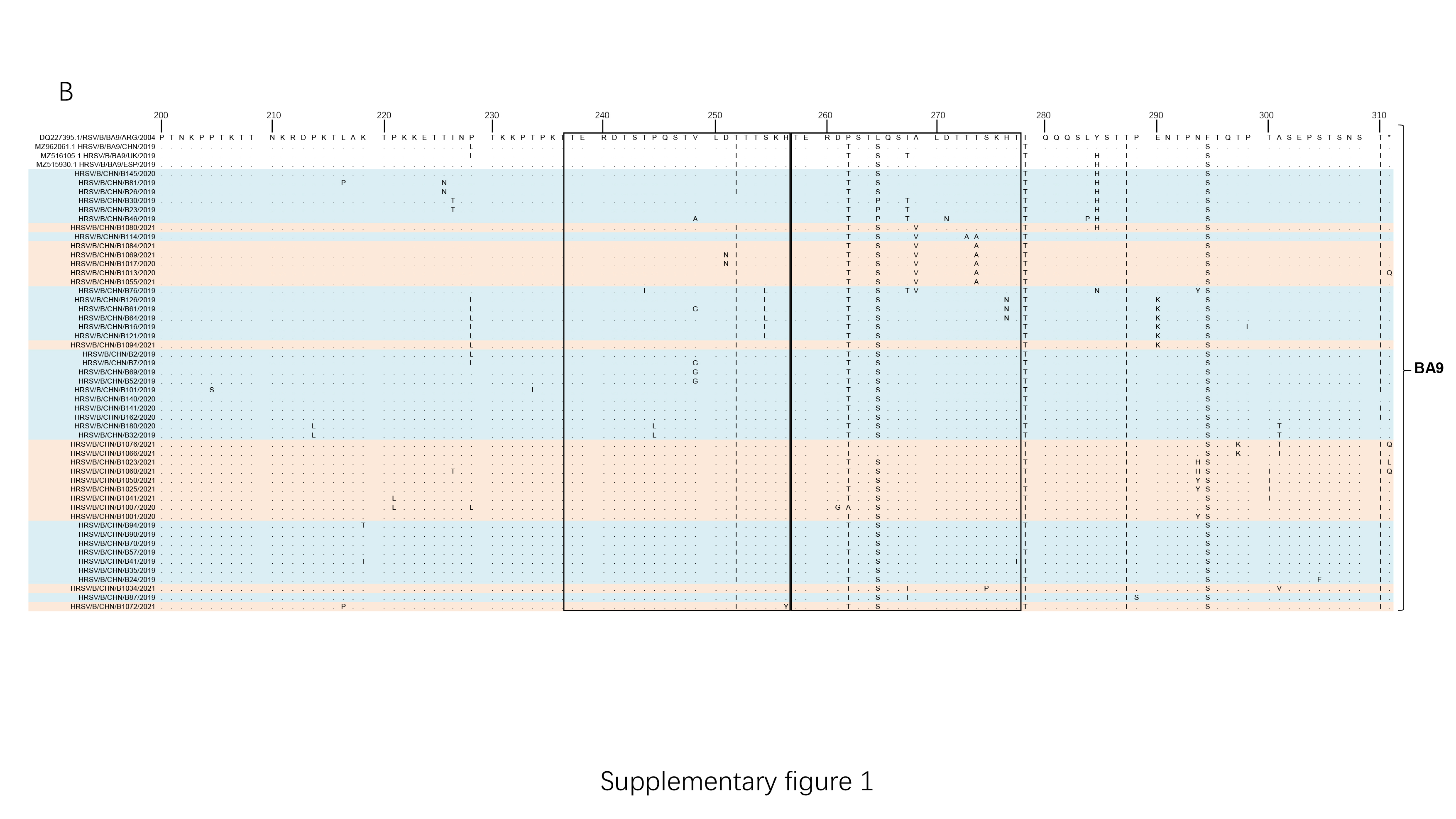


**Supplementary Figure 1. G protein sequences alignment of RSV ON1 and BA9.**

**A** The HVR2 in the C-terminal of G-protein sequences (200–322) of RSV A ON1 strains in our study was displayed, corresponding to the prototype ON1 strain ON67-1210A (Accession no. JN257693) and other closely related strains. **B** The HVR2 of G-protein sequences (200–311) of RSV B BA9 derived in the study were aligned with the prototype BA9 strain (Accession no. Q227395) and other reference strains. The duplicate regions are framed by rectangles. The RSV strains collected before and during COVID-19 are covered with blue and orange shades, respectively.

**Supplementary Table 1. Accession numbers of the sequences of HVR2 of the G gene derived in this study for phylogenetic analyses.**

| RSV genotype | GenBank accession numbers |
| --- | --- |
| RSV A ON1 | ON707406 ON707407 ON707408 ON707409 ON707410 ON707411 ON707412 ON707413 ON707414 ON707415 ON707416 ON707417 ON707418 ON707419 ON707420 ON707421 ON707422 ON707423 ON707424 ON707425 ON707426 |
| RSV B BA9 | ON707432 ON707433 ON707434 ON707435 ON707436 ON707437 ON707438 ON707439 ON707440 ON707441 ON707442 ON707443 ON707444 ON707445 ON707446 ON707447 ON707448 ON707449 ON707450 ON707451 ON707452 |

| Variables | | Univariate analysis | |  | Multivariate analysis | |
| --- | --- | --- | --- | --- | --- | --- |
|  |  | RR (95% CI) | *p*-value |  | RR (95% CI) | *p*-value |
| Before/During COVID-19 | |  |  |  |  |  |
|  | Before | Reference |  |  |  |  |
|  | During | 0.203(0.075-0.546) | 0.002* |  | 0.322(0.109-0.948) | 0.040* |
| Season at onset | |  |  |  |  |  |
|  | Not Summer | Reference |  |  |  |  |
|  | Summer | 0.486(0.114-2.074) | 0.33 |  |  |  |
| RSV subtype | |  |  |  |  |  |
|  | A | Reference |  |  |  |  |
|  | B | 0.815(0.321-2.066) | 0.67 |  |  |  |
| Gender | |  |  |  |  |  |
|  | Male | Reference |  |  |  |  |
|  | Female | 0.542(0.214-1.374) | 0.20 |  |  |  |
| Age |  |  |  |  |  |  |
|  | 0-5m | Reference |  |  |  |  |
|  | 6m-18y | 0.7(0.288-1.701) | 0.43 |  |  |  |
| Gestational age at birth, weeks | |  |  |  |  |  |
|  | ≥37 | Reference |  |  |  |  |
|  | <37 | 2.492(0.982-6.320) | 0.06 |  | 1.673(0.619-4.525) | 0.31 |
| Co-infection with other respiratory viruses | |  |  |  |  |  |
|  | No | Reference |  |  |  |  |
|  | Yes | 0.540(0.184-1.588) | 0.26 |  |  |  |
| Underlying diseases | |  |  |  |  |  |
|  | No | Reference |  |  |  |  |
|  | Yes | 2.656(1.047-6.737) | 0.040* |  | 1.905(0.727-4.990) | 0.19 |
| Neutrophils, ×10^9/L | |  |  |  |  |  |
|  | <1.5 | Reference |  |  |  |  |
|  | 1.5-4 | 2.848(0.631-12.850) | 0.17 |  | 2.440(0.535-11.130) | 0.25 |
|  | >4 | 5.043(1.071-23.750) | 0.041* |  | 3.049(0.602-15.452) | 0.18 |
| PCT, ng/dL | |  |  |  |  |  |
|  | <0.3 | Reference |  |  |  |  |
|  | 0.3-0.5 | - |  |  |  |  |
|  | >0.5 | 1.393(0.530-3.666) | 0.50 |  |  |  |
| Cr, μmol/l | |  |  |  |  |  |
|  | <18 | Reference |  |  |  |  |
|  | 18-24 | 1.144(0.369-3.546) | 0.82 |  |  |  |
|  | >24 | 0.622(0.176-2.206) | 0.46 |  |  |  |

**Supplementary Table 2. Univariate and multivariate analysis of risk factors for severe LRTI cases in children infected with RSV.**

RR, relative risk; CI, confidence interval.

**P*<0.05
